# Current Perspectives on Prevention of Mother-to-Child Transmission of Syphilis

**DOI:** 10.7759/cureus.525

**Published:** 2016-03-09

**Authors:** Eleonor G Lago

**Affiliations:** 1 School of Medicine, Pontifícia Universidade Católica do Rio Grande do Sul - PUCRS; 2 Edipucrs University Publisher, Pontifícia Universidade Católica do Rio Grande do Sul - PUCRS

**Keywords:** congenital syphilis, syphilis serodiagnosis, sexually transmitted diseases, vertical transmission, antenatal care, pregnancy, disease prevention

## Abstract

This article aims to provide an update on the prevention of mother-to-child transmission of syphilis by drawing upon some important basic concepts and reviewing the most recent literature on the diagnosis and treatment of syphilis in pregnancy. New technologies, such as automated and point-of-care immunologic tests, are shifting some paradigms, which will certainly be further investigated in the forthcoming years. This is the time to carefully evaluate traditional as well as new strategies to prevent congenital syphilis. Adverse outcomes of mother-to-child transmission of syphilis can be prevented with antenatal screening and penicillin therapy, which proved to have an excellent cost-benefit ratio even in populations with a low prevalence of syphilis. However, syphilis epidemiology is influenced by socioeconomic and cultural factors, and major challenges are faced by poor and developing countries in which the severity of the problem is extremely alarming. On the other hand, the emergence of new technologies has raised doubts about the best algorithm to be used when proper laboratory resources are available. Conditions are quite heterogeneous across populations, and some procedures should not be generalized while there is no evidence that supports some changes and while in-depth studies about local conditions are not conducted. Official organizations need to be alert in order to avoid isolated decisions and ensure that evidence-based guidelines be used in the management of syphilis in pregnancy.

## Introduction and background

The term, congenital syphilis, has been traditionally used to describe the effects of syphilis on pregnancy. However, in 2012, the World Health Organization (WHO) experts suggested that, whenever possible, the term mother-to-child transmission (MTCT) of syphilis be used so as to highlight all the possible adverse outcomes of the disease, which include spontaneous abortion, stillbirth, prematurity, clinical manifestations of congenital syphilis, infant death, and late sequelae [1]. Recent studies, whether evidence-based retrospective analyses with large samples [2-5] or a prospective follow-up of infants born to mothers with gestational syphilis [6], corroborate the association of syphilis with these adverse gestational outcomes and with severe disease in the infected infants.

As penicillin has a high efficacy against *Treponema pallidum* infection in pregnancy, MTCT of syphilis could virtually be eliminated. Several strategies have been proposed to achieve this goal, which is, however, far from being attained in most populations; syphilis in pregnancy continues to be a major global problem in the second decade of the 21st century [1-10]. More women have syphilis than HIV infection and, taking into account only untreated pregnant women, MTCT of syphilis (nearly 100%) is higher than that of HIV (around 30%). While some global disease elimination programs are successful, the elimination of MTCT of syphilis so far has not achieved such results. However, this will become possible if interventions targeting vulnerable groups are implemented [11-12].

Over 90% of people infected with syphilis live in low- and middle-income countries, but the problem also affects developed countries [1, 13-14]. Nevertheless, there has been occasional uncertainty over whether antenatal screening for syphilis should be maintained in populations in which this sexually transmitted infection is rare. Additionally, the emergence of new technologies, such as automated immunologic tests, has raised doubts about the best algorithm to be used when proper laboratory conditions are available.

This article aims to provide an update on the prevention of MTCT of syphilis by drawing upon some important basic concepts and reviewing the most recent literature on the diagnosis and treatment of syphilis in pregnancy as well as programs targeted at the elimination of MTCT of syphilis. The review does not include diagnosis or management of congenital syphilis in neonates. PubMed, PubMed Central, Scopus, and Virtual Health Library databases were searched using the combined keywords, syphilis and pregnancy, and focusing initially on articles published in 2014 and 2015. Official publications by WHO, Pan-American Health Organization (PAHO), Centers for Disease Control and Prevention (CDC), United Nations Children’s Fund (UNICEF), Australasian Society for Infectious Diseases (ASID), International Union against Sexually Transmitted Infections-Europe (IUSTI-Europe), and Brazilian Ministry of Health were also reviewed, as well as recent books and articles which, regardless of their date of publication, remain important to the full understanding of the current perspectives on the prevention of MTCT of syphilis.

## Review

### Antenatal screening for syphilis

Syphilis may go clinically unnoticed, especially in women, in whom even the primary stage may not have visible symptoms. It is paramount that pregnant women be examined many times during antenatal care (ANC) in order to detect any suggestive finding on physical examination; however, both the detection and diagnosis of syphilis are based chiefly on routine serological tests. ANC should be initiated within the first 12 weeks of gestation, and the first screening test for syphilis must be performed as soon as possible in pregnancy. In populations with a high prevalence of syphilis, the screening test must be repeated at the beginning of the third trimester (28th week of gestation) and at delivery [15-16]. In some populations, the detection of syphilis only at delivery is quite common, mainly if the woman did not have a proper ANC. Some authors recommend one more test one month after delivery to detect acquired infections at the very end of pregnancy [6, 17-19].

Traditional Algorithm

**Figure 1:**
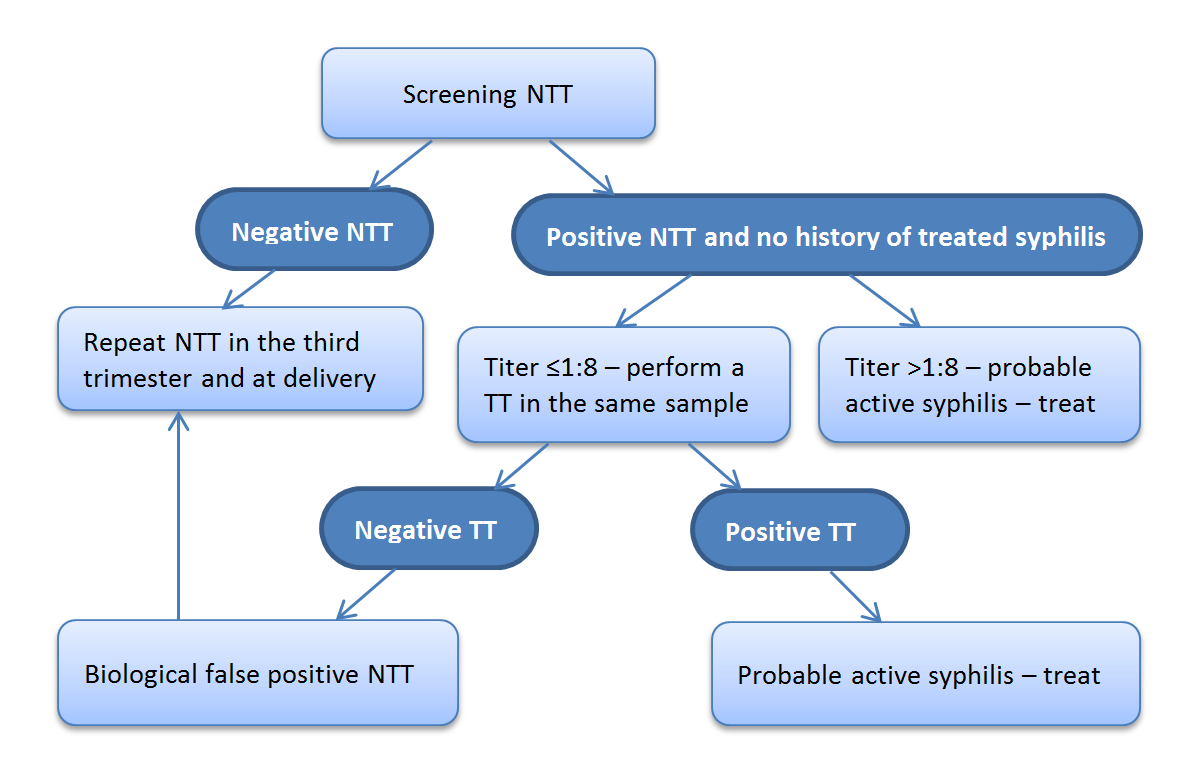
Traditional algorithm for diagnosis of syphilis in pregnancy, in which a nontreponemal test is used for the initial screening. Pregnant women with previously treated syphilis require a different approach (see text). NTT: nontreponemal test; TT: treponemal test

According to the traditional algorithm, a nontreponemal test (NTT) is used as a screening test and a treponemal test (TT) as a confirmatory one (Figure 1). A pregnant woman with probable active syphilis (PAS) is identified by the positive results in both types of tests (NTT and TT).

Conventional screening for syphilis uses two types of NTT: either the rapid plasma reagin (RPR) or the venereal disease research laboratory (VDRL). These widely used tests detect antibodies against cardiolipin, a cell membrane component of mammals and of *T. pallidum*. The advantage of a VDRL is that it can be done in the cerebrospinal fluid, unlike RPR. As reactivity may occur around the fourth week after contamination, its sensitivity in primary syphilis is about 75%, compared to almost 100% in secondary and congenital syphilis. The advantages of RPR is that it provides a slightly higher sensitivity in the primary stage and can be processed faster. This is quite useful for testing of pregnant women since it can be performed during an antenatal visit, thus, allowing for the prompt onset of treatment, if necessary. In several countries, this is the most widely used test at ANC clinics. An important characteristic of an NTT is that it allows assessing treatment efficiency, given that after adequate treatment, its titer decreases and is often negative within a few months. NTT may yield false-positive results and, therefore, must be confirmed by a TT, which is more specific. Numerous conditions, such as collagen diseases and malignancies, may produce positive NTT results, which, in these cases, are regarded as biological false positives, unlike the false positives caused by technical laboratory problems. Pregnancy itself is a frequent cause for biological false positive NTT results, with rates ranging from 5% to 20% of pregnant or puerperal women. While, in these cases, NTT titers are usually low (≤ 1:4), in rare situations (as with the use of intravenous drugs), the titers can be high even in the absence of syphilis. Conversely, NTT may give false negative results due to the prozone reaction, in which high antibody titers saturate the antigen, interfering with the antigen-antibody complex formation. Currently, the prozone reaction is rare because laboratories prevent it by testing any sample using a dilution of at least 1:8 [16, 20-23].

Conventional TT use lyophilized *T. pallidum *antigens, as in the fluorescent treponemal antibody absorption (FTA-ABS), or a *T. pallidum* lysate, as in the treponemal specific microhemagglutination test (MHA-TP). MHA-TP is now replaced by the *Treponema pallidum* particle agglutination (TP-PA), considered of superior quality owing to particle homogeneity. In the conventional algorithm, TT is not used for the screening because, in addition to being more expensive and technically more complex than NTT, they lose specificity if used in the general population, yielding 1 to 2% of false positives. On the other hand, when used as confirmatory tests in a population previously screened by NTT, their specificity reaches approximately 100%. This is due to the fact that the predictive value of a diagnostic test is determined not only by its sensitivity and specificity but also by the prevalence of the disease in the population to be tested. TT are not useful in evaluating treatment efficiency or in distinguishing active infection from a previous one, as they often remain reactive forever, even after the infection has been cured, except in special cases when treatment for acquired syphilis is initiated very early [18, 20, 23].

Techniques for the detection of IgM, either by immunoassay or Western blot, are not accurate enough to determine the risk for congenital syphilis, regardless of whether they are performed in the mother or in the infant [15, 23-24].

Reverse Algorithm

**Figure 2:**
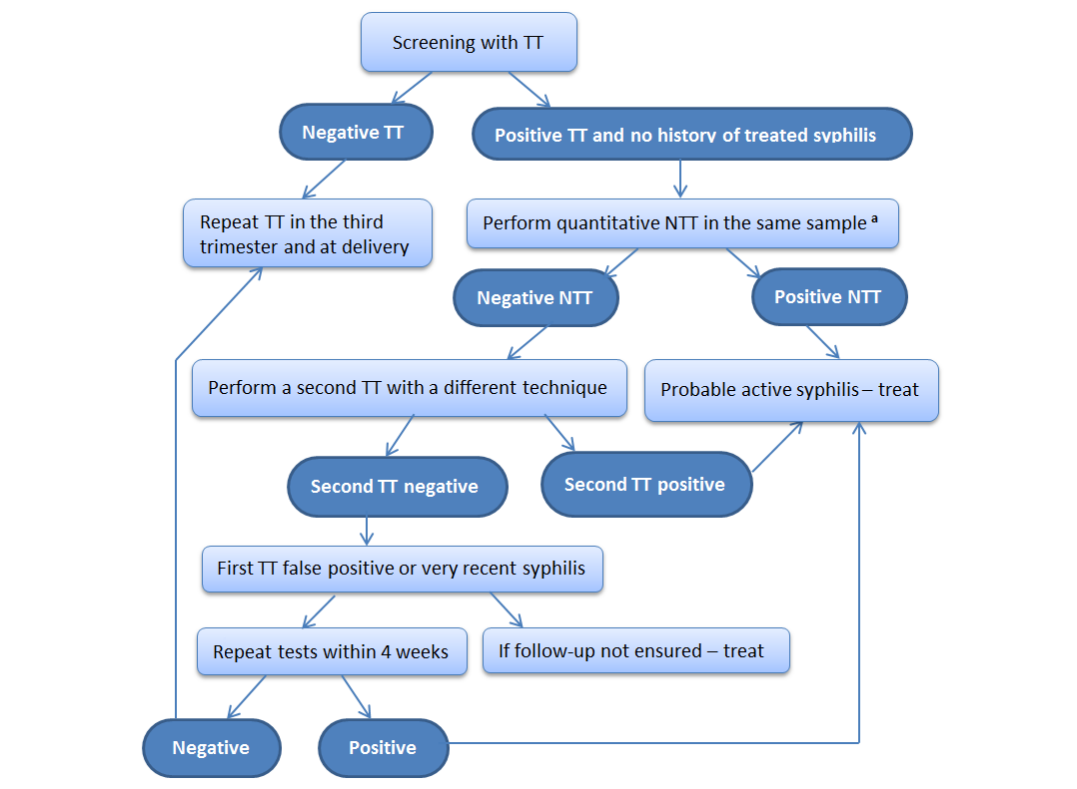
Reverse algorithm for diagnosis of syphilis in pregnancy, in which a treponemal test is used for the initial screening. Pregnant women with previously treated syphilis require a different approach (see text). TT: treponemal test; NTT: nontreponemal test ^a^ A quantitative NTT titer is important for identifying active syphilis, monitoring treatment efficiency, and to compare with the newborn's NTT at delivery. In some settings where the prevalence of syphilis is high and follow-up is uncertain, the pregnant woman may receive treatment without waiting for a confirmatory TT.

The development of automated tests, which utilize recombinant treponemal antigens such as the enzyme immunoassay (EIA), chemiluminescence immunoassay (CLIA), multiplex flow immunoassay (MFI), or microbead immunoassay (MBIA), has led some laboratories to employ a reverse algorithm, i.e., to use TT for screening, followed by a NTT in positive cases. If the NTT is also positive, it indicates PAS and the individual must be treated (Figure 2). It is important to perform the NTT not only to identify PAS but also to have a starting point for monitoring treatment efficiency. Additionally, in pregnant women, a titer at delivery is important for comparison with the neonate’s NTT titer. Any situation in which the presence or not of active syphilis is questioned necessitates a careful assessment of the patient’s clinical picture and history [15, 25-26].

The reverse algorithm may produce discordant results in which the TT is positive and NTT is negative; this does not occur in the conventional approach where TT is only performed in cases of a positive NTT. In the presence of a negative NTT, TT positive results suggest a previous infection, but in the absence of prior treatment for syphilis, the possibilities are false positive results, recent syphilis, or tertiary syphilis. Whenever a discordant result is obtained, a confirmatory TT should be performed using a different technique. TP-PA, which is more specific and probably more sensitive than FTA-ABS, is the most recommended test for confirmation of discordant results. The confirmatory test should be performed preferably in the same sample. Other TT tests were also evaluated and worked out well as confirmatory tests [15, 23, 27-28].

According to the 2015 CDC guidelines for the screening and diagnosis of syphilis among pregnant women using the reverse algorithm, in discordant cases (positive TT and negative NTT) in which the second TT is positive, previously treated syphilis or very recent infection is confirmed. There is no need to treat pregnant women whose treatment was adequate and documented; however, pregnant women without a history of treatment should be treated. If the second TT is negative, the first one is more likely to have been a false positive, especially in a low-risk woman with no history of previous syphilis and without any clinical evidence, whose partner also does not have clinical or serological evidence of syphilis. If it is possible to follow this woman, the tests should be repeated within four weeks. If the confirmatory TT and the NTT remain negative, treatment is not necessary. If follow-up is not ensured, the woman should be treated according to the stage of acquired syphilis [15, 29-30].

For Europe, the International Union against Sexually Transmitted Infections/Europe guidelines recommends another version of the reverse algorithm. When screening includes only TT and the result is positive, a TT different from the first one is indicated. If the confirmatory TT is positive, treatment is recommended, and in this case, an NTT should be performed to control treatment response. The guidelines do not make it clear what the recommendation is if the second TT is negative [31].

Regardless of the algorithm used, it is important that the laboratory follows a clear routine. Whenever a confirmatory test is necessary, this test should be done in the same sample before disclosing the result to the patient or to the physician. The results should be made available only after the whole set is ready [25].

Some advantages of the reverse algorithm include the use of automated techniques that process more samples simultaneously and higher sensitivity in detecting late latent syphilis, in which the NTT could be negative. The sensitivity of automated TT is comparable to that of conventional TT. Regarding sensitivity in primary syphilis, results vary widely as, in some studies, the sensitivity of automated TT was lower than that of NTT but similar or higher in others [25, 27, 32].

Notwithstanding, the use of this reverse sequence has some limitations, including elevation of costs due to the necessity of new equipment and additional confirmatory tests, greater likelihood of false positive results, more unnecessary treatment, and more complex diagnostic decisions, which comprise several combinations of positive and negative tests. The cost-benefit ratios vary according to the prevalence of syphilis. Insofar as these limitations are concerned, the use of reverse algorithm has become widely disseminated. This is due not only because of automation efficiency in laboratories with a high volume of tests but also because of the possible application of the technique to rapid tests, whose results can be obtained during medical appointments. These tests will be discussed further ahead [23, 25, 27, 33].

Morshed and Singh, in a comprehensive review of diagnostic tests for syphilis, presented the recent trends in Canada [23]. According to them,

"*The decision to use the conventional or reverse algorithm should be made based on a combination of the local syphilis prevalence, the expected (clinical and laboratory) workload, the requirement for automation, and the available budget. Since it is anticipated that more laboratories will continue to use or switch to newer algorithms for syphilis testing, laboratorians and clinicians will have to become more comfortable with interpreting test results when testing starts with a treponemal test*" [23].

Point-of-Care Treponemal Rapid Tests

If either conventional or new automated tests were used appropriately in all pregnant women and management was based on the test results, they would be enough to practically eradicate congenital syphilis. However, in addition to the fact that the management of syphilis among pregnant women relies on several factors (which will be discussed further ahead), laboratory tests cannot always be applied, as they require technical and logistic resources that might not be available. A meeting, jointly promoted by the WHO Sexually Transmitted Diseases Diagnostics Initiative and by the Welcome Trust, was hosted by the Bill and Melinda Gates Foundation in 2002. The meeting aimed to forge a global alliance to stimulate the development and translation of more efficient tests for the diagnosis of STIs in developing countries. Such tests should be easily performed during medical appointments and should meet the ASSURED criteria: A=affordable, S=sensitive, S=specific, U=user-friendly (simple to perform, uses non-invasive specimens), R=rapid and robust, E=equipment-free, and D=delivered (accessible to end-users) [21, 34-36].

Currently, several programs for the control of HIV and syphilis have already introduced rapid diagnostic tests known as point-of-care (POC), most of which utilize immunochromatographic strips. These tests can be done during ANC visits, not requiring the laboratory infrastructure necessary for conventional tests, and can be performed in whole blood, serum, or plasma samples. They are usually carried out in finger-stick blood samples, are simple to perform, and their results are easy to interpret, being available in approximately 20 minutes. These tests have good sensitivity and specificity, comparable to those of conventional NTT and TT [10, 35, 37-38].

While making the management of gestational syphilis easier in certain populations, rapid POC TT has some limitations. As they detect treponemal antibodies, their results need to be confirmed by an NTT to avoid unnecessary treatments in patients without active syphilis. Nonetheless, these tests were designed for resourceless settings with a high prevalence of syphilis. In these cases, given the high morbidity of MTCT of syphilis, every pregnant woman with any positive results (TT or NTT in any titer), should be treated. The risks associated with untreated gestational syphilis remarkably outweigh the risk of overtreatment [15, 21, 39].

The prevention of congenital syphilis depends on factors other than the type of test to be used, mainly on the access of pregnant women to ANC as well as treatment availability. A forthcoming section in this review will address the strategies for the control of MTCT of syphilis, and some other aspects of rapid POC tests will be discussed.

Filter Paper Dried-Blood Rapid Tests

Tests carried out in dried blood spots on filter paper, widely used in neonatal screening, have been introduced for the antenatal screening of infectious diseases. This method is chiefly useful where access to laboratory facilities is difficult since dry blood from a finger prick can be taken to the laboratory under environmental conditions. In the case of syphilis, EIA is used to detect treponemal antibodies of both classes (IgG and IgM). These tests have good sensitivity and specificity and have been used in some settings in Brazil as routine antenatal screening, including the investigation of other infections in the same blood sample [40-43].

Smit, et al. [44] reviewed the literature on the use of filter paper samples for the diagnosis of tropical diseases and concluded that the technique allows for an accessible, robust, sensitive, and specific diagnosis in remote settings and could be an important tool for health worldwide. However, the authors warn that existing studies lack standardization of materials, collection methods, and statistical analysis and that research with more rigorous methods are needed so that the actual role of filter paper samples in the management of syphilis and other infections among pregnant women may be evaluated [44].

Dual Point-of-Care Treponemal/Nontreponemal Tests

An immunochromatographic strip test is being developed, which can identify the presence of treponemal and nontreponemal antibodies, allowing for the diagnosis of previous or active disease, or biological false-positive results. When tested in Atlanta, this dual treponemal/nontreponemal test proved to have good sensitivity and specificity [45]. A rapid test that could identify the presence or absence of PAS in a single sample would be ideal as a POC test in ANC could restrict the number of pregnant women who need to undergo additional diagnostic tests or who are submitted to unnecessary treatment [23, 46]. The sensitivity and specificity of the dual POC test were tested in Chinese volunteers and yielded good results [47]. In Australia, the test was applied to laboratory-stored blood samples and compared against reference tests; it had good concordance, except for the cases of treated syphilis, in which concordance was equal to 27.5%. Among previous infections, the dual test identified 49.8% as probable active syphilis and 22.8% as non-syphilis [48]. To our knowledge, the dual POC test has not been assessed in pregnant women yet.

Electronic Readers

Electronic readers use digital imaging technology to transmit data through a smartphone or another device containing smartphone technology. According to Wedderburn, et al., this technology could provide automated and standardized results, enabling (1) quality assurance in the correct conduct of testing and interpretation of results; (2) valid and timely surveillance data; and (3) supply chain management and availability of critical supplies [49]. No studies have evaluated the use of readers in the prevention of MTCT of HIV and syphilis; the authors suggest that field studies are necessary to assess how these readers can be adapted to the ANC setting [49].

### Treatment of syphilis in pregnant women

At present, there is only one antibiotic indicated for the treatment of pregnant women with syphilis: parenteral penicillin G. Penicillin has been used for over 70 years and no case of resistance of *T. pallidum* to this drug has been reported. Treatment of syphilis requires the presence of treponemicidal levels of the antibiotic for long periods because of the slow rate of reproduction of *T. pallidum*. The slow-release benzathine penicillin G (BPG) is highly efficacious against syphilis, including in pregnant women [15, 50-52].

Penicillin Doses

Recently, Clement, et al. carried out a systematic review and found a grade of evidence A for the treatment of recent syphilis (duration of less than one year) using a dose of 2,400,000 international units (IU) of BPG [52]. This is the usual dose recommended for pregnant women with primary, secondary, or early latent syphilis. For instance, the 2015 CDC guidelines [15] and the chapter by Kollman and Dobson in the 2016 edition of Remington and Klein’s book [16] state that the treatment of pregnant women should follow the usual recommendation for the specific stage of acquired syphilis. Nonetheless, these guidelines also warn that several specialists would rather give an additional dose to pregnant women. Failure of a single dose of 2,400,000 IU to prevent MTCT of syphilis in pregnant women with primary, secondary, or early latent syphilis has been reported [53-55]. Therefore, some experts recommend treatment of recent syphilis in pregnant women with at least one weekly dose of 2,400,000 IU of BPG for two weeks, totaling 4,800,000 IU. As pregnant women who received at least two doses of BPG in general initiated treatment earlier, it is possible that the better results of this regimen are related to the earlier treatment rather than to the higher dose. Thus, the best dose of BPG for pregnant women with recent syphilis is still controversial [15-16, 50-52, 56]. 

A greater risk of treatment failure associated with syphilis-related fetal ultrasound abnormalities has been repeatedly reported. Based on this information, some authors suggest that additional doses of BPG should be given to the pregnant woman until there is a resolution of the fetal ultrasound findings or delivery. However, there is insufficient evidence to date regarding this issue, and a therapeutic schedule has not been determined. Intravenous penicillin administration should be avoided in these cases due to the higher risk of complications caused by the Jarisch-Herxheimer reaction, especially in the presence of hydrops fetalis (see the specific topic on Jarisch-Herxheimer reaction further ahead) [15-16, 50-51].

According to the systematic review of Clement, et al. [52], the treatment recommended for the late stages of syphilis still has a grade of evidence C. It is believed that *T. pallidum* replicates more slowly at later stages, and, therefore, it is necessary to maintain penicillin administration for a longer time. Hence, according to these authors and others, despite the grade C level of evidence, the three weekly doses of 2,400,000 IU of BPG, totaling 7,200,000 IU, are still the recommended treatment of syphilis with over one year of duration, unknown duration, and also during pregnancy [15, 51-52, 57].

Given that patients often do not return for the second or third dose, some have adopted a single dose as appropriate for the treatment of pregnant women with syphilis, regardless of the disease's stage. Studies demonstrated that at least one dose of 2,400,000 IU of BPG given before the 28th week of gestation was quite efficacious in reducing the adverse outcomes of MTCT of syphilis. For example, Watson-Jones, et al. [58] showed that pregnant women who tested positive for syphilis and were treated with a single dose of BPG had the same number of adverse gestational outcomes than those who had tested negative for the disease. Authors who advocate the single-dose regimen also argue that the better results obtained with three doses might be due to other possible interventions in a more comprehensive ANC, and not specifically to the higher number of doses of penicillin [50, 58-59].

Studies that assessed the clinical picture of neonates with congenital syphilis describe that the presence of some kind of penicillin therapy, even when the dose is considered to be insufficient and/or when NTT titers do not decrease significantly, actually protect the infant from more severe disease. These studies reveal that virtually all the mothers of neonates that were severely ill at delivery had not received any dose of penicillin. Nevertheless, inadequate maternal treatment defines a case of congenital syphilis, and asymptomatic neonates born to these mothers must be investigated and treated [6, 15-17, 57, 60].

Time of Treatment

The time at which treatment is initiated during the gestational period is of paramount importance, as the efficiency in avoiding possible adverse outcomes of MTCT of syphilis is better when penicillin is given before the 21^st^ week of gestation, or at least before the 28^th^ week [51, 59]. However, even if syphilis is detected only after fetal involvement, treatment could still be efficacious. Resolution of fetal abnormalities detected by ultrasound examination has been reported after the treatment of syphilis in pregnant women [61]. The indicated dose of BPG when fetal signs are found was discussed above.

Treatment failures resulting in congenital syphilis have also been described among pregnant women when treatment was given less than 30 days before delivery. Placental adaptations at the end of gestation alter the pharmacokinetics of penicillin, lowering its serum levels. For that reason, or simply because there is not enough time for a therapeutic effect on the fetus, treatment completed after the 30 days prior to delivery is regarded as inadequate, and the neonate should be classified as a case of congenital syphilis, investigated, and treated accordingly [16, 55].

Allergy to Penicillin

No other antibiotic but penicillin has proven to be efficient in preventing MTCT of syphilis. Therefore, pregnant women who are allergic to this antibiotic should be desensitized and treated with adequate doses of BPG [15-16, 18, 56]. Allergic reactions are very rare. A systematic review included 3,466,780 syphilis patients treated with penicillin, of whom 1,244 were pregnant women. No severe reactions were described, whereas four deaths occurred in the general population as a consequence of adverse reactions to penicillin (incidence 0-0.003%) [62].

Jarisch-Herxheimer Reaction

The Jarisch-Herxheimer reaction is an acute condition consisting of fever, headache, myalgia, and other symptoms within two to 24 hours after starting treatment for syphilis. It occurs in about 40% of the pregnant women treated for syphilis, especially those treated in the second half of pregnancy, who may have contractions and decelerations of fetal heart rate. The woman should be advised to seek medical attention if she experiences fever, decreased fetal movements, or regular contractions in the first 24 hours following treatment. Fetuses with hydrops may have fetal distress soon after maternal treatment; therefore, hospitalization is recommended for fetal monitoring in these cases. However, the possibility of early labor induction or fetal distress should not prevent or delay treatment for syphilis [15, 31, 51, 55].

Treatment of Partners

It is essential that pregnant women and their sex partners be treated concomitantly, given the high risk of reinfection. The guidelines adopted in Brazil [57] include a partner with syphilis not treated as one of the criteria for the definition of cases of congenital syphilis. Even guidelines that do not include this criterion recommend that sex partners be treated, regardless of having negative tests for syphilis [15-16]. However, there are some shortcomings associated with this approach since some pregnant women are reluctant to tell their partners about their status and also because some of the partners refuse to undergo treatment [63-65].

Follow-up of Pregnant Women after Treatment

After proper treatment for primary or secondary syphilis, NTT titers decrease by at least four times (two dilutions) within three or four months, and eight times (four dilutions) within six to eight months. In most patients, the titers become negative after one year. In the presence of reinfection, the titers increase. After the treatment of a pregnant woman, NTT should be repeated every month, and the proper response criterion is a fourfold reduction in titers. The control must be done with the same type of test used initially. If the expected decrease in titers does not occur until before delivery, the case must be regarded as non-responsive to treatment. A fact that may hinder a prompt fourfold decrease in titers is that they may increase a little bit shortly after treatment. A study revealed that in 20% of the patients the RPR titers increased within two weeks after treatment, decreasing thereafter. If the baseline titer is obtained at the beginning of treatment, it might seem there was no serological response unless a longer time period is allowed. The serological response in HIV-positive patients can be slower [15, 20, 66-67].

The persistence of clinical signs, a fourfold increase in NTT titers or their failure to decrease by four times after six months, indicates therapeutic failure or reinfection. In these cases, treatment should be repeated with three weekly doses of 2,400,000 IU of BPG. A cerebrospinal fluid test and an HIV screening should be performed in the woman. If a pregnant woman is diagnosed with neurosyphilis, treatment should be administered accordingly. It should be recalled that pregnant women treated early are at higher risk of reinfection [15-16].

Some patients have low titers (up to 1:2) even after they are cured – the so-called "serofast" people. Thus, persistently low NTT titers do not mean active infection and do not require repeating treatment, providing that the previous one was adequate. Even among serofast patients, the NTT titers increase in the case of reinfection. A pregnant woman with proper and well-documented treatment and with a persistently low NTT titer should be followed up closely and regularly retested. If the titer remains low and stable until delivery, the case can be regarded as adequate previous treatment [14-16, 20, 52].

It is important that any treatment of pregnant women be properly recorded. In settings where no communication exists among the several health sectors, a chart containing the results of all tests and treatments performed during ANC ought to be carried by the woman. Ideally, this chart should contain a specific space where the health professional can record each dose of penicillin given. Undocumented treatment is a frequent cause for defining a case of congenital syphilis, and this misdiagnosis could be avoided [6]. In addition to treatment documentation, it is paramount that the serological status between one pregnancy and the other be recorded so that a serofast woman is not considered to have active syphilis. A recent study carried out in Brazil indicated that in subsequent pregnancies of women who had been treated for syphilis in their previous pregnancy, there might be a larger number of neonates defined as cases of congenital syphilis even if they are not actually infected, which may be due to failures in treatment records and follow-up between the previous and present pregnancies [65]. In the presence of positive syphilis tests, without any information about the previous serological status and without treatment records, the pregnant or parturient woman is considered to have PAS, and the neonate must be managed and notified as a case of congenital syphilis [15, 60, 65].

### Control strategies for preventing mother-to-child transmission of syphilis

Owing to the difficulty in controlling the incidence of infection in the general population, strategies for the prevention of the severe consequences of MTCT of syphilis are targeted mainly at pregnant women. Syphilis epidemiology is influenced by socioeconomic and cultural factors that hinder the disease control. These major challenges are faced by poor and developing countries in which the severity of the problem is extremely alarming. Recent studies on the cost-benefit ratio have focused mainly on the poor populations of countries with low to intermediate socioeconomic levels, taking into account the impact related to MTCT of syphilis in terms of disability-adjusted life years. All studies suggest that ANC-integrated programs for syphilis are potentially cost-effective, considering the minimum quality criteria [68-71]. Moreover, studies demonstrated that this strategy has been highly cost-effective, even in developed countries and in populations with a low prevalence of syphilis. Therefore, the screening for syphilis is currently recommended at all ANC levels, including pregnant women from a good socioeconomic background [15, 18, 23, 72-73].

The most important risk factor for congenital syphilis is the absence or inadequacy of ANC, and almost all the maternal characteristics identified as risk factors (eg, use of illicit drugs, poverty, and poor education) interfere with proper ANC. Empirical evidence of how to improve access to ANC in each community is necessary because it is known that late access to ANC increases the risk for all the adverse effects of syphilis in pregnancy. About 20% of pregnant women in low- and middle-income countries do not have access to any ANC [1-2, 6-9, 68, 74]. In addition, the screening and treatment of syphilis in women who undergo ANC are quite variable and actually low in some populations. The lowest rates are observed in Sub-Saharan Africa where it is below 50% in most countries, although a rate as high as 95% has been reported. In most of Latin America and Asia, coverage ranges between 50 and 90%, whereas in European countries and in China, the rate exceeds 95%. Even within the same countries, coverage rates may be different depending on the region and on the different population groups [1, 7, 60]. Thus, efforts should be made to improve the coverage of ANC as an essential step towards eliminating MTCT of syphilis.

**Figure 3:**
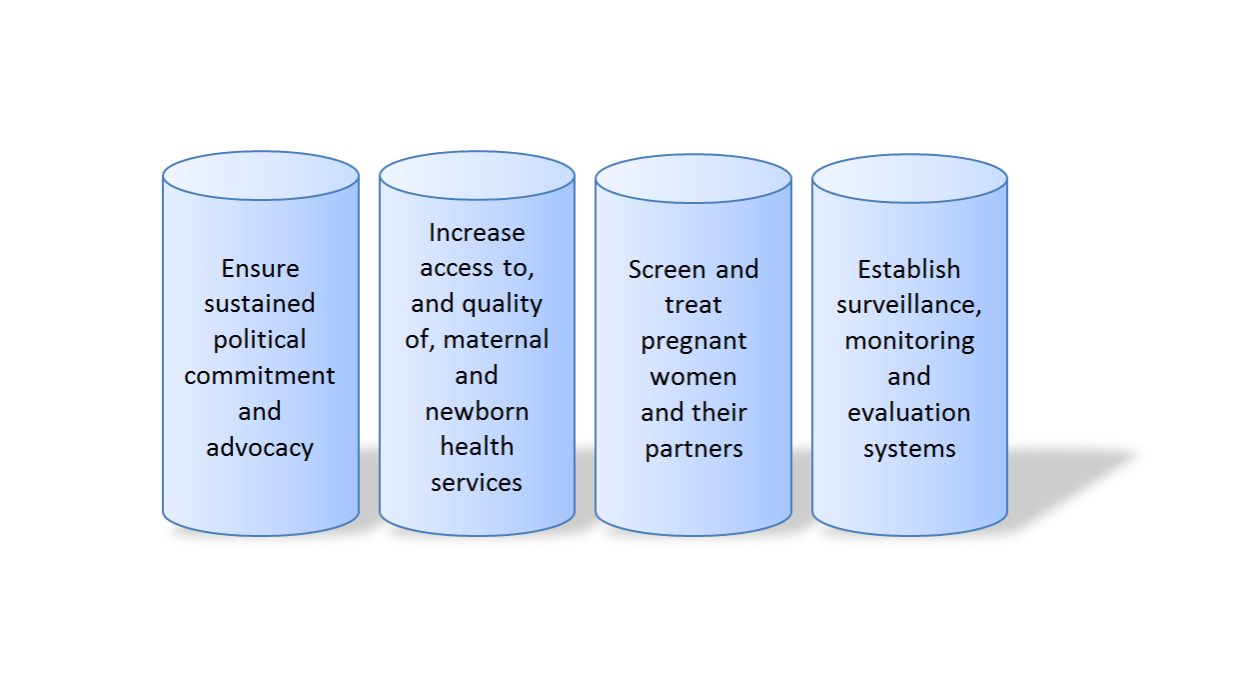
The four pillars that represent the strategy of the World Health Organization for elimination of congenital syphilis. Ref. [78-79]

In the 1990s, the PAHO and some countries, such as Brazil, had already launched programs for the eradication of congenital syphilis for Latin America and the Caribbean, acknowledging the great burden of MTCT of syphilis on the region [11, 75-76]. In 2013, the PAHO renewed its strategic plan [77]. The WHO’s global strategic plan for the elimination of congenital syphilis was launched in 2007. This plan establishes that the initiatives be adapted to each country; be integrated into other strategies, such as sexually transmitted infections/HIV programs, ANC programs, and mother-infant health; respect individual rights; and collaborate with institutions at various levels. Addressing these challenges, the WHO proposes four pillars of a strategy to eliminate congenital syphilis for countries to adopt, adapt, and implement (Figure 3) [78-79].

**Figure 4:**
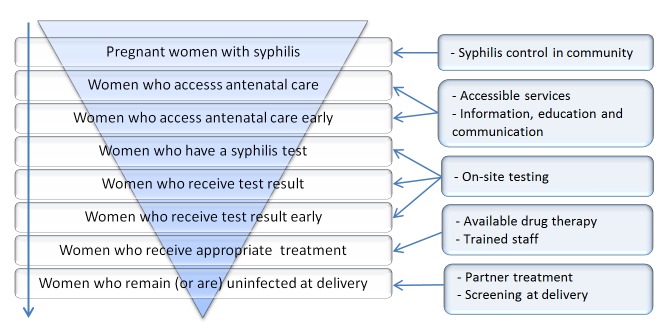
Stages to be followed in the prevention of mother-to-child transmission of syphilis. Each bar on the inverted pyramid represents a subgroup of women from the bar above. In order for prevention to be efficient, each stage requires a specific intervention (bars on the right) [72, 78].

Using the image of an inverted pyramid, WHO illustrates the stages that ought to be followed in the prevention of congenital syphilis. The wider stage of the pyramid comprises the whole group of pregnant women with syphilis and the levels get narrower as they go through each of the stages (Figure 4) [72, 78].

Potentially, the problems in several stages of this inverted pyramid can be overcome with the use of rapid POC tests, as previously described herein. However, many are the challenges that lie ahead, as these technologies, although of great value, are not capable of completely solving the problem of MTCT of syphilis. Recent studies carried out mainly in African countries show that with the advent of POC tests, several centers that had not been using syphilis screening in ANC started to do so. Nevertheless, this did not always mean that positive pregnant women received at least one dose of penicillin. Some centers that did relatively well by using conventional NTT screening reduced their screening rates after the introduction of the program. The advantages of rapid tests may be hindered by poor access to health centers and limited availability of resources (personnel, equipment, and medications), in addition to poor organization and effectiveness of clinical practice due to lack of knowledge, motivation, and supervision. Some authors suggest that it might be more productive to correct occasional failures in the existing programs than to replace them with new programs. For example, in settings where NTTs are being used for screening, rapid TT could be introduced as confirmatory tests instead of changing the screening procedures [10, 38, 80].

As stated by Larson, et al. [81],

"*while rapid syphilis tests are very good at finding the needle in a haystack, such testing only provides benefits if patients testing positive are actually treated (with at least one dose of penicillin). Otherwise, these rapid tests allow health workers to find the needle in the haystack, but the needle is then thrown back into the haystack*" [81].

A recommended strategy, which has already been implemented in many countries, concerns the integration between the prevention of HIV infection and other sexually transmitted infections. In ANC, the objective of integration is to take advantage of the existing framework for the prevention of MTCT of HIV.

In 2004, Valderrama [11] commented that

"*the major concern about the HIV pandemics had obfuscated the concern about syphilis*" [11].

In 2013, Hawkes [7] pointed out that

"*the recent global push for dual elimination of both HIV and syphilis has raised the political profile of syphilis in many parts of the world*" [7].

In the region of the Americas, the "Strategy and Plan of Action for the Elimination of Mother-to-Child Transmission of HIV and Congenital Syphilis in the Americas by 2015" was approved in 2010. PAHO and UNICEF have developed strategies for advancing towards the elimination of MTCT of HIV and syphilis. Great progress has been made since then in Latin America and the Caribbean. Five countries have met the target goals for elimination of MTCT of HIV, and 11 countries have reached those goals for syphilis [77].

In 2013, the WHO and the PATH (Program for Appropriate Technology in Health) initiated a project called Dual Testing for the Elimination of Congenital Syphilis. Methods that investigate antibodies against *T. pallidum* and HIV in the same sample and with the same equipment (dual POC tests HIV/syphilis) have been tested in African and Latin American countries and have shown good sensitivity and specificity. These methods will probably facilitate the integration of programs for the prevention of MTCT, ensuring that all pregnant women tested for HIV will also be tested for syphilis [36, 82-84].

Studies conducted in Africa and in Asia reported an increase in the screening for syphilis after integration with the screening for HIV in ANC without compromising the efficiency of HIV screening programs. In order for preventive measures to be efficient, it is necessary that ANC has good quality and be used by all women. These programs often use POC immunochromatographic strip tests, but some continue to use conventional NTT screening. In general, the screening coverage and the treatment of syphilis in ANC were enhanced. Nonetheless, in some programs, the HIV screening coverage was larger than that for syphilis [19, 64, 81, 85-89].

In a recent systematic review, Swartzendruber, et al. [90] selected articles carried out in eight low- and middle-income Asian, African, and South American countries that adopted integrated programs for the prevention of MTCT of HIV and syphilis and concluded that these programs increase the screening rates of both infections. Nevertheless, as also pointed out by other authors, supplies of high-quality commodities were seen as an implementation barrier and ensuring adequate training (performing tests, interpreting results, maintaining records, and managing stock) was cited by healthcare workers as a challenge in the context of frequent staff transfers and high staff turnover [90].

## Conclusions

Congenital syphilis and all the adverse outcomes of MTCT of syphilis can be prevented with antenatal screening and penicillin therapy, which have been used for decades and proved to have an excellent cost-benefit ratio, even in populations with a low prevalence of this infection. Currently, new technologies, such as automated immunologic tests, are shifting some paradigms, which will certainly be further investigated in the forthcoming years.

Unfortunately, in some socioeconomically underprivileged populations and in inaccessible places with scarce resources where the prevalence of *T. pallidum* infection is usually very high, syphilis in pregnancy still has unacceptably devastating consequences. To face this problem, international organizations have been investing in the development of new tests that are inexpensive and easily applied, as well as supporting strategies that allow diagnosing and treating the largest possible number of pregnant women. Recent research has shown that, under many circumstances, good results have been obtained with these innovative programs, often by integrating the prevention of MTCT of syphilis and HIV. However, despite such effort, the goals for the elimination of congenital syphilis set by these organizations have not been fully achieved. Access to ANC, screening coverage, and timely treatment are still challenges that must be overcome.

This is the time to carefully evaluate traditional as well as new strategies to prevent congenital syphilis. Conditions are quite heterogeneous across populations, and some procedures should not be generalized while there is no evidence that supports some changes and while in-depth studies about local conditions are not conducted. On the other hand, official organizations need to be alert in order to avoid isolated decisions and ensure that evidence-based guidelines continue to be used in the management of syphilis in pregnancy.

## Appendices

### Abbreviations

**ANC**: antenatal care; **ASID**: Australasian Society for Infectious Diseases; **BPG**: benzathine penicillin G; **CDC**: Centers for Disease Control and Prevention; **CLIA**: chemiluminescence-immunoassay; **EIA**: enzyme-immunoassay; **FTA-ABS**: fluorescent treponemal antibody absorbed​; **HIV**: human immunodeficiency virus; **IU**: international units; **IUSTI**: International Union against Sexually Transmitted Infections; **MBIA**: microbead immunoassays; **MFI**: multiplex flow immunoassay; **MHA-TP**: treponemal specific microhemagglutination​; **MTCT**: mother-to-child transmission; **NTT**: nontreponemal test; **PAHO**: Pan-American Health Organization; **PAS**: probable active syphilis; **POC**: point-of-care; **T. pallidum**: *Tr**eponema pallidum; ***TP-PA**:*Tr**epo**nema pallidum *particle agglutination; **TT**: treponemal test; **UNICEF**: United Nations Children's Fund; **VDRL**: Venereal Disease Research Laboratory​; **WHO**: World Health Organization
